# Diving behavior of the reef manta ray (*Mobula alfredi*) in New Caledonia: More frequent and deeper night-time diving to 672 meters

**DOI:** 10.1371/journal.pone.0228815

**Published:** 2020-03-18

**Authors:** Hugo Lassauce, Olivier Chateau, Mark V. Erdmann, Laurent Wantiez

**Affiliations:** 1 ISEA, University of New Caledonia, Nouméa, New Caledonia; 2 Conservation International New-Caledonia, Noumea, New Caledonia; 3 The Manta Trust, Corscombe, Dorchester, United Kingdom; 4 Laboratory of Marine Biology and Ecology, Aquarium des Lagons, Nouméa, New Caledonia; 5 Conservation International Asia-Pacific Field Division, University of Auckland, Auckland, New Zealand; Hawaii Pacific University, UNITED STATES

## Abstract

The interest in reef manta rays (*Mobula alfredi*) from the scientific community is growing in reaction to the major decline of populations around the world. Studies have highlighted the need to further investigate the spatial ecology of this species to inform conservation and management initiatives. Here we briefly report the results from nine SPLASH10-F-321A pop-off satellite archival tags (PSAT-tags) deployed in New Caledonia that recorded the world’s deepest known dives for reef manta rays. All tagged individuals performed dives exceeding 300 m in depth, with a maximum depth recorded of 672 ± 4 m. Diel comparisons revealed that most of the deepest dives occurred during night-time. We hypothesize this deep-diving behaviour is employed to access important food resources at these depths during the night and may also indicate that zooplankton abundance in the surface waters surrounding New Caledonian coral reefs is insufficient to sustain these megafauna. These results add new information on the habitat use of this species in a region where manta behaviour has not previously been studied, and increase the known depth range of *M*. *alfredi* by more than 200 m.

## Introduction

Reef manta rays (*Mobula alfredi*), are declining worldwide, in large part due to fishing pressure for their gill rakers [1,2]. Despite significant advances in our knowledge and understanding of this species in the past decade [3], more detailed information on the biology and the ecology of this species throughout its range is urgently needed [4]. Specifically, data on spatiotemporal dynamics and habitat use are necessary to develop concrete management plans and conservation actions [4] to prevent further declines of reef manta rays, now listed as “vulnerable” on the IUCN Red-List [5]. Satellite telemetry using pop-up satellite archival tags (PSAT tags) is one of the most effective methods to investigate fine scale horizontal and vertical movements and habitat use in manta rays [4,6,7–9], but until now there have been no such studies conducted in New Caledonian waters.

As planktivores, manta rays spend a major part of their time feeding or searching for foraging grounds [3,10–13]. Manta ray aggregations have been observed and monitored in multiple locations in tropical and sub-tropical waters around the world [3,12,14,16,17]. Seasonal or long-term presence of the species on a particular site is often associated with enhanced local productivity and increased food availability. For instance, seasonal migrations were found to be correlated with monsoonal shifts in the Indian Ocean [12,17]. As opportunistic feeders, manta rays are capable of undertaking relatively large-scale movements between productive areas (up to 750 km) [3,4,8,13–17]. Some studies have shown that reef manta rays are also able to explore substantial depths (up to 432 m), presumably to feed on deeper zooplankton and other food resources [8,10,18–20]. These foraging strategies remain unclear and more detailed information on this behaviour and the associated drivers are needed.

In New Caledonia, reef manta rays are not targeted by fishing, but have a highly fragmented distribution due to the specificity of their food resources and preferred habitat [8,13,16,21,22]. Environmental processes and conditions shape the distribution and the abundance of their zooplankton prey [21–24]. Nutrient enrichment is known to be the primary factor of phytoplankton proliferation, causing a subsequent increase in zooplankton abundance. Eutrophication benefit the development of phytoplankton upon which zooplankton feed [25,26]. Processes such as coastal upwellings and river run-off are both important sources of nutrient enrichment of coastal waters [26–28]. These processes, combined with tidal currents and bathymetry can support dense zooplankton concentrations and favourable feeding grounds for filter feeders such as *Mobula alfredi* [20]. Massive feeding aggregation of hundreds of reef mantas have been observed targeting such dense zooplankton aggregations in the Maldives [29,30] and occasionally in the southern reaches of the Great Barrier Reef [22]. In New Caledonia, manta feeding grounds seem to be scattered, with aggregations never exceeding a dozen individuals (Lassauce, pers. obs.).

This short communication presents the first data collected on the diving behaviour of reef manta rays in New Caledonia. These data reveal an unexpected outstanding feature: the unique depths and high number of deep dives, which considerably extend the known depth range for *Mobula alfredi*.

## Material and methods

### Ethic Statement

The tagging was conducted with authorizations from the Southern Province (permit no: 34584) and the Northern Province (permit no: 609011–33) of New Caledonia. In the Loyalty Islands Province, no permit was required by the competent authorities, though permission of the local customary representatives was granted.

### Study area

**Fig 1:**
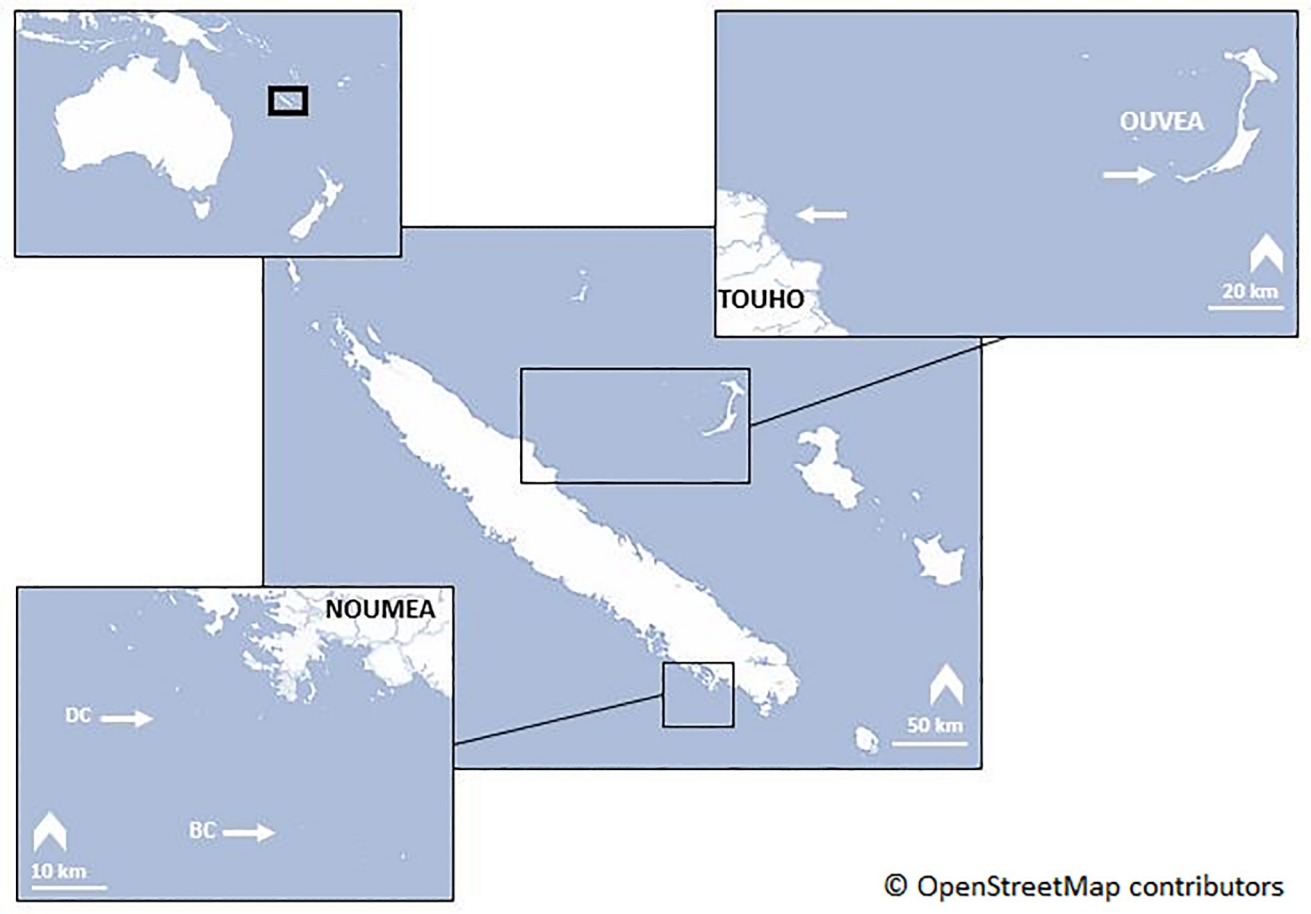
Tagging locations of *Mobula alfredi*. Arrows indicate tagging locations in Boulari channel (BC) and Dumbea channel (DC) in Noumea (n = 7), Touho (n = 3) and Ouvea (n = 1). Source: OpenStreetMap contributors.

A total of eleven tags were deployed on *M*. *alfredi* at three different locations in New Caledonia, an archipelagic nation consisting of a main island and three smaller islands off the east coast known as the Loyalty Islands (Fig 1).

One manta ray was tagged in Ouvea Island (20°43'S, 166°23'E) on the 4^th^ of December 2015 (tagging depth = 10 m). Seven individuals were tagged in two channels of the barrier reef surrounding the Main Island, Boulari channel (BC, 22°29'S, 166°26'E) and Dumbea channel (DC, 22°21’S, 166°15'E) between the 31^st^ of January and the 2^nd^ of February 2017 (tagging depth range = 5–15 m). Three tags were deployed in Touho channel (location undisclosed) between the 27^th^ and the 28^th^ of November 2018 (tagging depth range = 5–15 m) (Fig 1). Apart from Dumbea channel where manta rays aggregate to feed near the surface, the other tagging sites are all manta ray cleaning stations [31].

### Tagging process

This study used SPLASH10-F-321A PSAT tags (Wildlife Computers Inc., Redmond, Washington, USA) coated with Propspeed^™^ silicone coating to prevent fouling during the deployment period. These tags are equipped with a Fastloc-GPS receiver, allowing locations to be recorded even when the tag only surfaces for a brief period of time (0.2 second). All tags were programmed to archive light level, depth and sea temperature every 30 seconds and detach from the animal after a maximum of 180 days. Data were summarized every 12 hours and transmitted to the Argos satellite system (www.argos-system.org). Periods of 12 hours were chosen to represent daytime (from 7 am to 7 pm) and night-time (from 7 pm to 7 am). The twilight times varied from 5:04 am to 06:31 am and 6:58 pm to 5:42 pm at the time of the earliest deployment (04/12) and the latest release (16/06), respectively (civil twilight times). The maximum variation of the twilight times within the range of recorded movements is 9 minutes (https://meteogram.fr). Since the tag settings do not allow the precision to be able to discriminate crepuscular periods, we defined the daylight period from 7 am to 7 pm to ensure dusk is always included in the daytime period and dawn is always included in the night-time data. Tags were deployed while scuba diving. The tags are tethered by a 30 cm stainless steel cable to a titanium dart-tip that is applied into the dorsal musculature of the animal with a pole spear. Before being tagged, each manta ray was identified using photo-identification (except for tag #167754), its sex and maturity was determined, and its size was estimated (disc width DW to the nearest 10 cm) (Table 1). Maturity was assessed based on the presence of fully developed claspers for male individuals and the observation of either mating scars or pregnancy for female individuals.

**Table 1:** Summary of satellite tag deployment information and characteristics of the nine PSAT-tagged reef manta rays in New-Caledonia that successfully transmitted data.

Manta ID	Sex	Estimated disc width (cm)	ARGOS PTT tag ID	Date of tagging	Site of tagging	Deployment duration (days)	Data transmitted (%)
CD-MA-0109	Male, mature	300	#140916	04/12/2015	Ouvea (20”43’S, 166”23’E)	80	79
CD-MA-0004	Female, mature	330	#167755	30/01/2017	Noumea, BC (22”29’S, 166”26’E)	54	86
CD-MA-0166	Female, mature	350	#163079	31/01/2017	Noumea DC (22”21’S, 166”15’E)	136	65
CD-MA-0167	Female, juvenile	240	#151348	31/01/2017	Noumea DC (22”21’S, 166”15’E)	49	64
CD-MA-0168	Male, juvenile	260	#151349	31/01/2017	Noumea DC (22”21’S, 166”15’E)	50	100
CD-MA-0000	Male, mature	330	#167754	31/01/2017	Noumea, BC (22”29’S, 166”26’E)	110	42
CD-MA-0036	Male, mature	300	#167756	01/02/2017	Noumea, BC (22”29’S, 166”26’E)	Failed	Failed
CD-MA-0170	Female, mature	400	#167757	02/02/2017	Noumea DC (22”21’S, 166”15’E)	174	100
CD-MA-0026	Female, mature	340	#162378	28/11/2018	Touho (undisclosed)	Failed	Failed
CD-MA-0051	Female, mature	330	#162379	28/11/2018	Touho (undisclosed)	3	87
CD-MA-0047	Male, mature	320	#162380	29/11/2018	Touho (undisclosed)	5	85

All tags were SPLASH10-F-321A Fastloc GPS tags (Wildlife Computers Inc., Redmond, Washington, USA). Disc widths were visually estimated to the nearest 10cm. CD-MA-0036 and CD-MA-0026 failed to transmit data.

### Data analysis

Depths are presented as means (± SD) of the maximum depths and as maximum (± maximum accuracy) observed depths per period (day/night) over the total deployment duration. Maximum accuracy varied from 4 to 50 m with an average of 8.8 ± 9.9 m (n = 1099 dives). For diel comparisons for each individual, a Welch’s t-test was used as a non-parametric test for samples with unequal variances and a Student t-test was used for samples with equal variances. A Levene’s test was used to test the homogeneity assumption. A Kolmogorov-Smirnov test was run to compare the overall distribution of the number of dives per depth range during the night and the day. A Pearson’s r test assessed the linear relationship between deployment duration and maximum depths recorded. Finally, a Fisher exact test evaluated the diel difference for each of these depth ranges. Temperature data are minimum temperatures recorded at corresponding depth readings within each 12-hour period over the total deployment duration of all tags.

## Results and discussion

**Fig 2:**
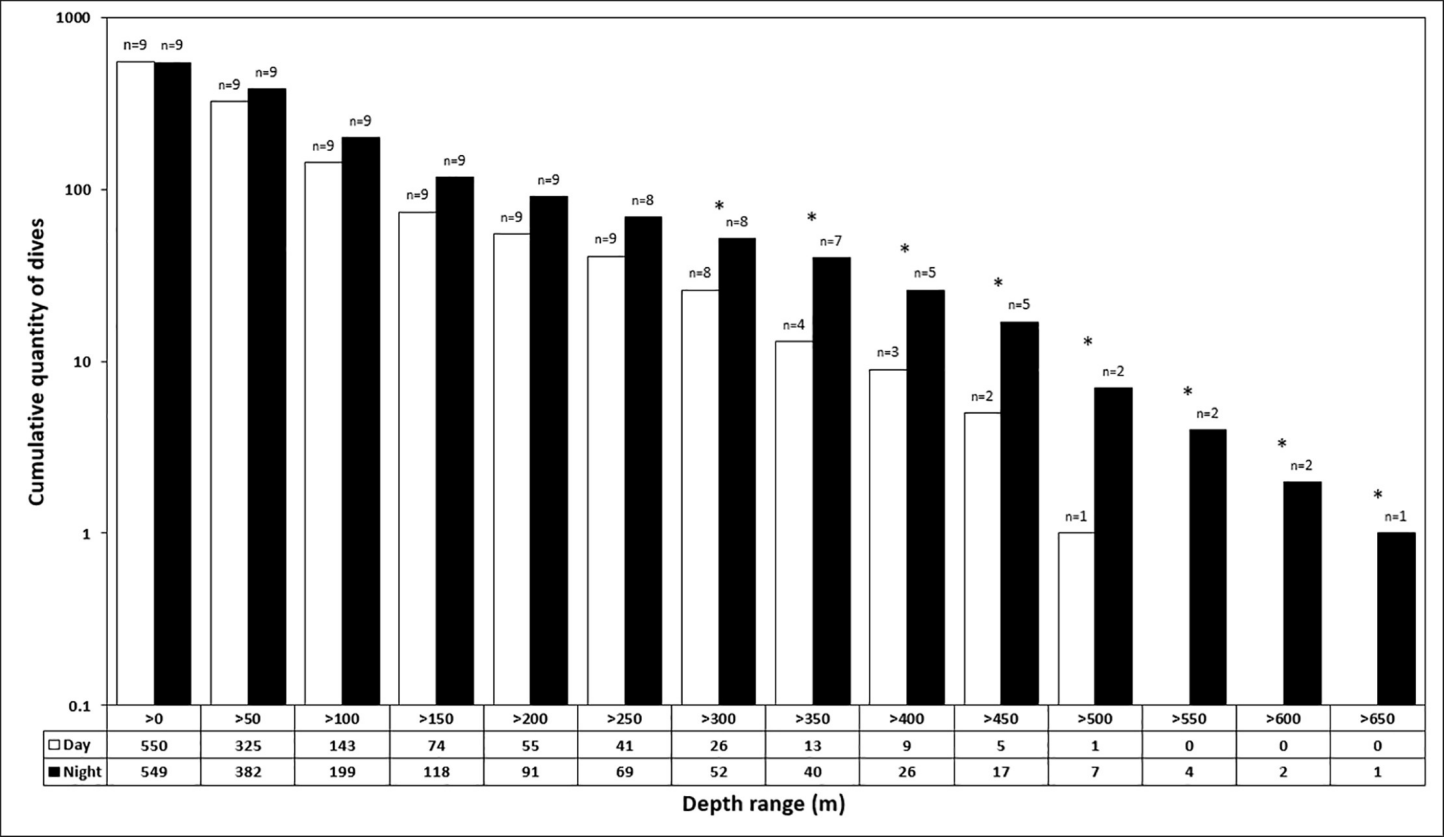
Comparison between day (white) and night (black) of the cumulative quantity of dives for all tagged reef manta rays at different depth ranges (m). n = total number of individuals recorded in each depth range. * indicates a significant difference (Fisher exact test, P < 0.05) between number of day and night dives in a given depth range.

Of the 11 tags deployed, two (#167756 and #162378) failed to transmit to the Argos system. The deployment duration of the functioning tags (n = 9) ranged from 3 to 174 days (73 ± 58 days). On average, 78 ± 19% of the data recorded by the tags was either transmitted by ARGOS satellite or downloaded from two tags recovered after deployment (Table 1). All nine individuals recorded dives deeper than 300 m (n = 78), and six of them performed dives deeper than 450 m (n = 22), including two exceptionally deep dives by two of the smaller tagged individuals (2.4 m female CD-MA-0167 and 3 m male CD-MA-0109) that reached maximum depths of 624 ± 4 m and 672 ± 4 m, respectively (Fig 2). This last dive extends the reported depth range for *M*. *alfredi* by more than 200 m, previously recorded as 432 m in the Red Sea [8]. A similar study in Indonesia using the same tags and tagging technique recorded a reef manta ray reaching a maximum depth of 624 ± 4 m in East Kalimantan (Erdmann, unpub.). In this study only 6 of the 30 tagged manta rays recorded dives deeper than 300 m, which indicate fewer deep dives compared to New Caledonia. In the Red Sea, 5 of the 7 tagged individuals dived deeper than 300 m [8] and none of the tracked manta rays in Eastern Australia reached these depths [7]. In New Caledonia, all individuals dived deeper than 300 m, representing 7.1% of all dives (n = 1099). The 200 m level was reached in 13% of all dives (n = 1099). The mean depth of all the dives was 103.1 ± 104.9 m (n = 1099) (Table 2). Maximum depths recorded were not correlated with deployment duration (Pearson’s r test, r = -0.19, n = 9, p > 0.5). The dives recorded by the New Caledonian mantas are thus both deeper in an absolute sense and more frequently exceeding the 200m mark than previously found in other parts of the world [7,8,32].

**Table 2:** Dive profiles between the nine PSAT-tagged reef manta rays.

Manta ID	Day Depth (m)		Night Depth (m)		% of dives with a maximum depth > 300m	Time at maximum depth during the deepest dive (min)
	Mean ± SD	Max. ± Max. Accuracy	Mean ± SD	Max. ± Max. Accuracy		
CD-MA-0109	176.9 ± 95.4	512 ± 4	217.9 ± 134	672 ± 4	21.8	2.2
CD-MA-0004	100.2 ± 54.3	344 ± 4	128 ± 77.9	472 ± 4	3.8	10.1
CD-MA-0166	71.9 ± 37.8 *	350 ± 50	105.6 ± 75.1 *	496 ± 4	5.8	10.8
CD-MA-0167	77.4 ± 55	464 ± 4	123.5 ± 115.6	624 ± 4	7.1	16.6
CD-MA-0168	95.8 ± 47.8 *	328 ± 4	146 ± 96.2 ±	384 ± 4	9	6.5
CD-MA-0000	79 ± 57.3	450 ± 50	87.8 ± 54.1	350 ± 50	6.7	13
CD-MA-0170	54.6 ± 32.4 *	304 ± 4	65.9 ± 38.8 *	224 ± 4	0.3	1.4
CD-MA-0051	118 ± 77	272 ± 4	192 ± 122.7	376 ± 4	14.3	26.6
CD-MA-0047	257.6 ± 87.7	360 ± 4	252.8 ± 140.2	480 ± 4	40	14.4

* indicates p < 0.05. Means are averages of the maximum depths recorded for each dive.

Difference in mean depths per individual between day and night were only significant for three manta rays. Regarding the diel comparison of the overall distribution of the number of dives per depth range, no significant difference (p > 0.05) was observed (Table 2).

Among all individuals, the number of deep dives (depth > 300 m) was only significantly larger at night (7 pm– 7 am) than during the day (7 am– 7 pm) (Fisher exact test, p = 0.010) (Fig 2). This behaviour could be explained by the nocturnal exploitation, at night, of demersal food sources, which has been observed in reef manta rays [4,18,19], oceanic manta rays [33], other mobulid species [34–36], as well as whale sharks [37]. In this study, all manta rays spent a relatively short amount of time at maximum depth during their absolute deepest dive. Bottom time during each manta’s absolute deepest dive averaged 11 ± 7 minutes, varying from 26.6 minutes at 376 ± 4 m to 1.4 minutes at 304 ± 4 m (Table 2). There was no significant correlation between maximum depth reached and the time spent at this depth (Pearson’s r test, r = - 0.06, n = 9, p > 0.5).

**Fig 3:**
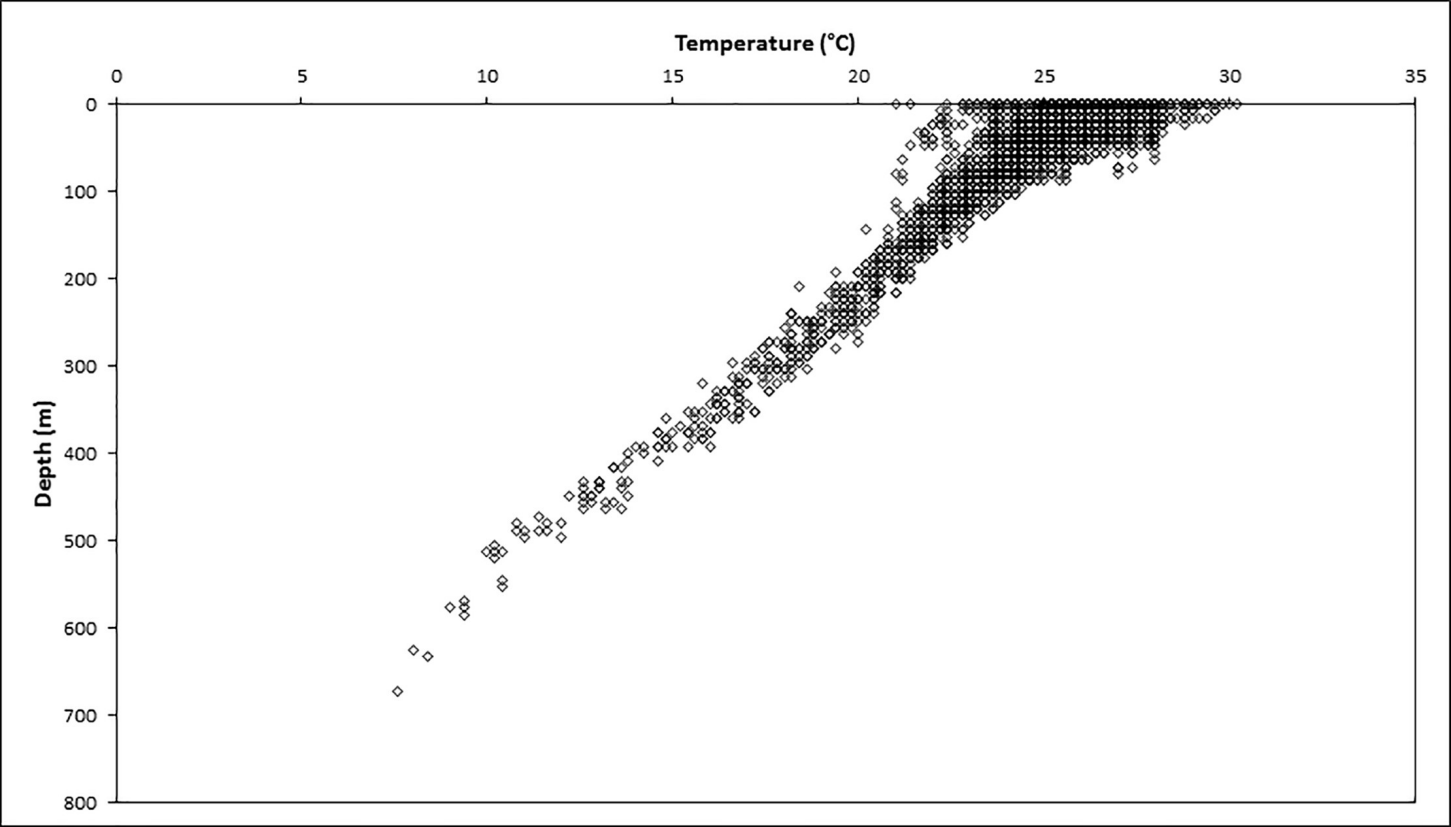
Relation between the minimum temperature (°C) at corresponding depth (m) measurements (n = 3820) during the deployment of all tags (n = 9).

Analysis of dive profiles can provide valuable information on diving behaviour [38]. Classification of dive profiles has been mainly conducted on air-breathing marine animals such as seabirds [39], sea turtles [40], or seals [41], as well as a few studies focused on predatory fish [42,43]. These analyses revealed two main patterns that have been associated with distinct behaviours. Dives with very short or no bottom time, called “V-Shaped” dives, are possible indicators of travelling and/or prey searching behaviour [38–42]. By comparison, U-shaped or square-shaped dives profiles with distinctively longer bottom times are thought to suggest foraging activities [38–42]. Asymmetrical V-shaped dives were described for reef manta rays by Braun et al. [8]. These authors suggested that short bottom times with relatively slow descents and faster ascents reflected an optimized travelling behaviour using gliding [8]. In this study, three manta rays showed this type of profile with very limited time spent at maximum depth during their deepest dive (1.4 min at 304 ± 4 m, 2.2 min at 672 ± 4 m and 6.5 min at 384 ± 4 m) (Table 2). While travelling and/or prey searching could be an explanation for these particular dives, additional data on the velocity during ascent and descent would be needed to test this hypothesis. On the other hand, our results show that six manta rays remained at maximum depths for more than 10 minutes. These dive patterns are more akin to U-shaped profiles, suggesting the exploitation of aggregated prey [38,42]. As fishes, manta rays diving is not limited by the ability to store oxygen, but more probably by the low temperatures at these depths. During dives, temperatures were always colder than 20°C below 300 m, with a minimum temperature of 7.6°C recorded at the maximum depth of 672 ± 4 m (Fig 3). Manta rays are poikilothermic species with an optimal thermal range from 20 to 26°C [3,7,32]. Previous studies have also shown that mobulid rays have the capacity to transmit warmth to the brain using a specific vesicular network in the pectoral fin that can function as a counter‐current heat exchanger [44]. Consequently, basking in warm shallow water prior to diving and active swimming during descent and ascent could be used to increase the body temperature. This mechanism would allow manta rays to produce enough heat to reach demersal food resources and feed for a relatively short amount of time despite the cold temperatures of these depths. This behaviour has been observed for other mobulid rays and other fish such as tunas and sharks [33–37,44–47]. In order to fully support this hypothesis, more detailed data on the dive profile of these manta rays are necessary to confirm rapid descent and slower ascent directly followed by an extended period of basking in warm shallow water. If this last hypothesis can be verified, the identification of such comportment for the reef manta rays of New Caledonia highlights the probable presence of important demersal food resources at depth, resulting in significant foraging success that presumably compensates for the energetic costs. The unusual depths reached and number of deep dives recorded suggest that foraging opportunities could be insufficient in the upper layer of the water column in New Caledonian waters, thereby forcing manta rays to explore deeper food resources. Detailed data on resource availability at varying depths and on the diet of manta rays in this region will help in determining the underlying drivers of their movements.

These preliminary results extend the global knowledge on the depth range and, more generally, the habitat use of *M*. *alfredi*. In this case, these data appear to support previous findings that prey at mesopelagic depths (from 200 m to 1000 m) [48] are valuable, if not indispensable, food resources for reef manta rays [10,18,19]. A comprehensive knowledge of the distribution and the habitat use of the reef mantas is necessary to inform conservation and fisheries management measures to ensure the long-term survival of the species [4]. Protective legislation has improved in recent years and numerous marine protected areas (MPAs) have been created throughout the known range of reef manta rays [49]; however, many of these MPAs are coastal in nature and do not extend into the deeper offshore waters used by reef mantas. As deepwater fisheries are increasingly exploiting the mesopelagic zone [50], our study highlights the importance of incorporating offshore waters and deep-water foraging grounds in manta conservation initiatives.

## Supporting information

S1 Fig Data. Supporting data.
(XLSX)
